# Passive seismological approaches for localizing near-surface fiber-optic cables with DAS

**DOI:** 10.1038/s41598-026-52485-9

**Published:** 2026-05-28

**Authors:** Georg Rümpker, Fabian Limberger, Abolfazl Komeazi

**Affiliations:** Institute of Geosciences, Goethe University Frankfurt, Altenhöferallee 1, 60438 Frankfurt, Germany

**Keywords:** Distributed acoustic sensing (DAS), Fiber-optic cables, Channel localization, Cable routing, Ambient noise cross-correlation, Array seismology, Passive seismology, Engineering, Natural hazards, Solid Earth sciences

## Abstract

Accurate knowledge of fiber-optic cable geometry is important for many applications of distributed acoustic sensing (DAS). However, the true positions of buried or installed fibers are often uncertain due to slack, bends, or deviations from documented routes. We present two passive, seismology-based approaches for cable localization that exploit information contained in DAS recordings. The approaches are evaluated based on synthetic tests under controlled conditions. Case A employs ambient noise cross-correlations with reference points to estimate relative travel times, whereas Case B uses the differential arrivals of plane waves from distant earthquakes with linearly independent slowness vectors. Both methods can be formulated using a least-squares approach that allows for the joint estimation of propagation velocity and geometry, thereby providing consistent solutions in the presence of noisy or uncertain travel-time measurements. Synthetic experiments show that cable positions can be recovered with an accuracy in the order of 100 m, even when apparent velocities are uncertain or the medium exhibits heterogeneity. The two methods provide independent geometric constraints that complement other sources of information on cable routing, although additional uncertainties are expected in field applications.

## Introduction

Fiber-optic cables are widely deployed for telecommunications, data transport, and environmental sensing, ranging in scales from urban networks to transoceanic links^1,2^. For applications such as maintenance, infrastructure monitoring, and geophysical observations, it is essential to know the precise location of the cable^3–6^. However, telecommunication fibers often include slack loops, bends, or deviations from trenching paths, while installation documentation often lacks the accuracy required for georeferencing^7–9^. As a result, although the general route of the cable may be known, the specific geometry and coupling vary along the line, and the linear distance along a cable cannot always be reliably mapped to geographic coordinates.

Conventional localization methods include tracer wires, detectable with surface-based electromagnetic tools (e.g^10^.), and optical time-domain reflectometry (OTDR), which measures distances to faults^11^. These approaches either require frequent surface access or provide no geographic coordinates, which limits their applicability to long buried or submerged cables (^12^ for a review).

Distributed acoustic sensing (DAS) provides an alternative to these conventional localization approaches by using standard optical fibers as dense seismic arrays (e.g^13^.). DAS interrogators send laser pulses and analyze the backscattered light to detect strain variations along the cable, enabling ground-motion monitoring at meter-scale spacing over tens of kilometers^14^. Most analyzes assume that channel positions are known a priori^15,16^, but this assumption is often violated when using “dark fiber” infrastructure not designed for geophysical purposes (i.e., unused telecommunication fiber-optic cables that are not actively transmitting data^7^). A few recent studies have explored ways to overcome this limitation by analyzing DAS recordings without relying on exact channel coordinates, for example by using blind near-field array processing to extract wavefield properties directly^17^. Nevertheless, for many seismological and monitoring applications, accurate georeferencing of the fiber remains essential^3,9^.

Field calibration by surface impacts or vehicle-towed taps can provide approximate positions, but these approaches are labor-intensive and impractical in many settings^18,19^. This highlights the need for alternative localization methods that exploit the continuous data already recorded by DAS.

In this paper, we introduce two passive, seismology-based approaches for fiber-optic cable localization. Case A exploits ambient noise cross-correlation between reference and unknown points, while Case B uses arrival times of transient plane waves with known linearly independent slowness vectors. Both methods can be extended to least-squares formulations when additional data are available, offering improved robustness and flexibility for cable localization. The two approaches developed here can provide independent geometric constraints from DAS recordings in situations where conventional survey information is incomplete or uncertain.

We present a proof-of-concept study for passive seismological approaches to DAS-based cable localization under controlled conditions. The analyses are based on synthetic wavefields that allow for a systematic evaluation of the proposed methods. While the synthetic tests include heterogeneous velocity structures and travel-time uncertainties, real-world DAS data are affected by additional complexities such as strong-small-scale heterogeneity, scattering, directional effects, and variable coupling conditions. These factors may influence signal coherence and travel-time estimation. Their implications, as well as potential methodological improvements, are discussed in the Discussion section.

## Methodologies

To use passive seismic recordings for cable localization, we develop two complementary approaches based on DAS. The first (Case A) uses ambient noise cross-correlation with reference points, while the second (Case B) exploits plane-wave arrivals from distant earthquakes. Both methods are used to convert travel-time measurements into geometric constraints on channel positions and can be extended to least-squares formulations that incorporate heterogeneous velocities and additional constraints.

### Case A: Ambient noise cross-correlation

**Fig. 1:**
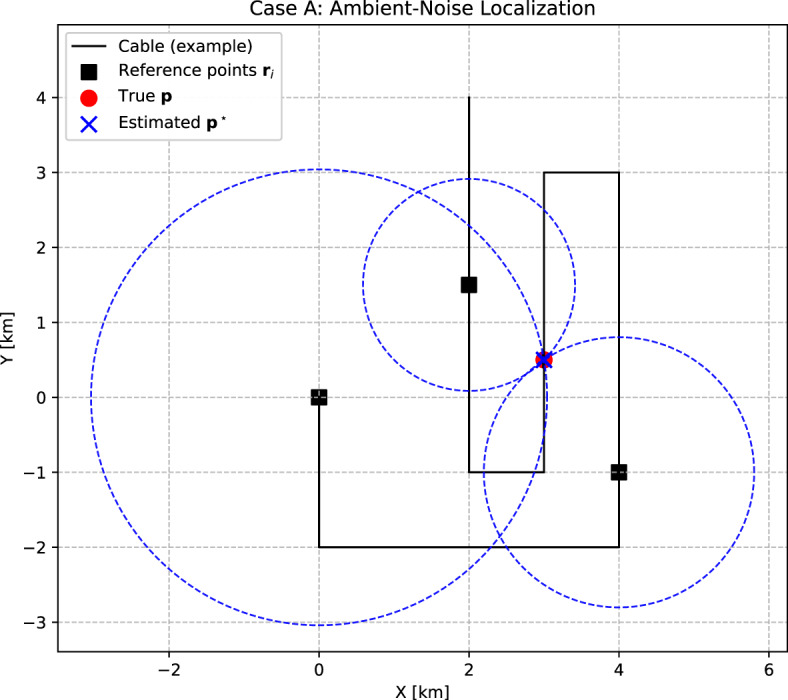
Localization of an unknown channel $\textbf{p}$ from ambient noise cross-correlation (Case A). The locations of three reference points $\textbf{r}_i$ are assumed to be known, while dashed circles represent the loci of possible positions defined by the measured travel times. Each circle radius is given by $R_i = v \, \tau _i$, where $v$ is the assumed constant propagation velocity and $\tau _i$ is the observed travel time from reference *i* to $\textbf{p}$. In the ideal case, the circles intersect at a single point corresponding to the true position; in practice, inconsistencies in the travel-time lead to a misfit, and the estimated position is obtained by a least-squares approach.

Cross-correlation of continuous seismic noise between two receivers provides an estimate of the empirical Green’s function, with coherent arrivals typically corresponding to surface (Rayleigh) waves^20^. This principle can be applied to localize an unknown point $\textbf{p}=(x,y)$ along a fiber-optic cable using at least three known reference points $\textbf{r}_i=(x_i,y_i)$, $i=1,2,3$ (Fig. 1). Cross-correlations between $\textbf{p}$ and the reference points yield relative travel times $\tau _i$, defined as positive lag times. The mean propagation velocity *v* of the relevant seismic waves can be estimated from travel times and known distances between reference points, or assumed from near-surface geology.

Assuming constant $v$, the distances satisfy

1 $$\begin{aligned} \Vert \textbf{p} - \textbf{r}_i\Vert = v\,\tau _i = R_i, \quad i=1,2,3. \end{aligned}$$

These equations define circles centered at $\textbf{r}_i$ with radii $R_i$, whose intersection gives the location of $\textbf{p}$. By squaring and subtracting one equation from the others, a linear system is obtained. For example, subtracting the equation for $i=1$ from those for $i=2,3$ yields

2 $$\begin{aligned} \begin{bmatrix} x_1 - x_2 & y_1 - y_2 \\ x_1 - x_3 & y_1 - y_3 \end{bmatrix} \begin{bmatrix} x \\ y \end{bmatrix} = \frac{1}{2} \begin{bmatrix} R_2^2 - R_1^2 + x_1^2 - x_2^2 + y_1^2 - y_2^2 \\ R_3^2 - R_1^2 + x_1^2 - x_3^2 + y_1^2 - y_3^2 \end{bmatrix}. \end{aligned}$$

This system has a unique solution provided that the reference points are not collinear. In practice, reference points should therefore be chosen such that they span a sufficiently wide angular range as seen from the unknown channel.

In the absence of errors, Eq. (2) yields a unique solution. However, when travel-time picks are noisy, the radii $R_i$ become inconsistent, and the circles do not intersect at a single point. In this case, the problem is most appropriately cast as a least-squares estimation of the location:

3 $$\begin{aligned} \textbf{p}^\star \;=\; \arg \min _{\textbf{p}} \sum _{i=1}^3 \left( \Vert \textbf{p}-\textbf{r}_i\Vert - v\,\tau _i \right) ^2. \end{aligned}$$

Here, $\textbf{p}^\star$ denotes the least-squares estimate of the channel location, i.e. the value of $\textbf{p}$ that minimises the misfit. The inclusion of additional reference points leads to an overdetermined system, and is recommended in practice (see next paragraph and examples in the Results section).

Because the residuals are nonlinear functions of the model parameters, iterative solvers are typically employed (e.g^21^.). We use the Levenberg–Marquardt algorithm to linearize the problem around a starting position, update the parameters, and repeat until convergence (e.g^22^.).

#### Inversion with unknown global velocity

If the uniform propagation velocity $v$ is not known a priori, it can be treated as an additional unknown and estimated jointly with the channel coordinates. In this case, it is advantageous to use all available measurements in a simultaneous inversion. Let $\textbf{r}_i=(x_i,y_i)$, $i=1,\dots ,N_\textrm{ref}$, denote the reference positions and $\textbf{p}_j=(x_j,y_j)$, $j=1,\dots ,N_\textrm{ch}$, the unknown channel locations. Then, the measured travel times $\tau _{ji}$ correspond to pseudo-distances $v\,\tau _{ji}$.

The joint inversion can be formulated as an alternating least-squares scheme. For fixed *v*, each channel position is updated by a Levenberg–Marquardt step,

4 $$\begin{aligned} \textbf{p}_j^{(k+1)} = \arg \min _{\textbf{p}_j} \sum _{i}\left( \Vert \textbf{p}_j-\textbf{r}_i\Vert -v^{(k)}\tau _{ji}\right) ^2, \end{aligned}$$

which is applied to all unknown channel positions $\textbf{p}_j$, each of which is updated independently from its travel-time constraints. The procedure is repeated for all channels within each iteration using the current global velocity estimate $v^{(k)}$.

For fixed positions, the velocity can be updated in closed form as

5 $$\begin{aligned} v^{(k+1)} = \frac{\sum _{j,i} \tau _{ji}\,\Vert \textbf{p}_j^{(k+1)}-\textbf{r}_i\Vert }{\sum _{j,i} \tau _{ji}^2}\, . \end{aligned}$$

This update arises directly from setting the derivative of the misfit with respect to $v$ to zero. The formula computes the propagation velocity that best fits all channel–reference pairs simultaneously. It is computationally efficient since it avoids a potentially more costly grid search.

### Case B: Plane-wave illumination

**Fig. 2:**
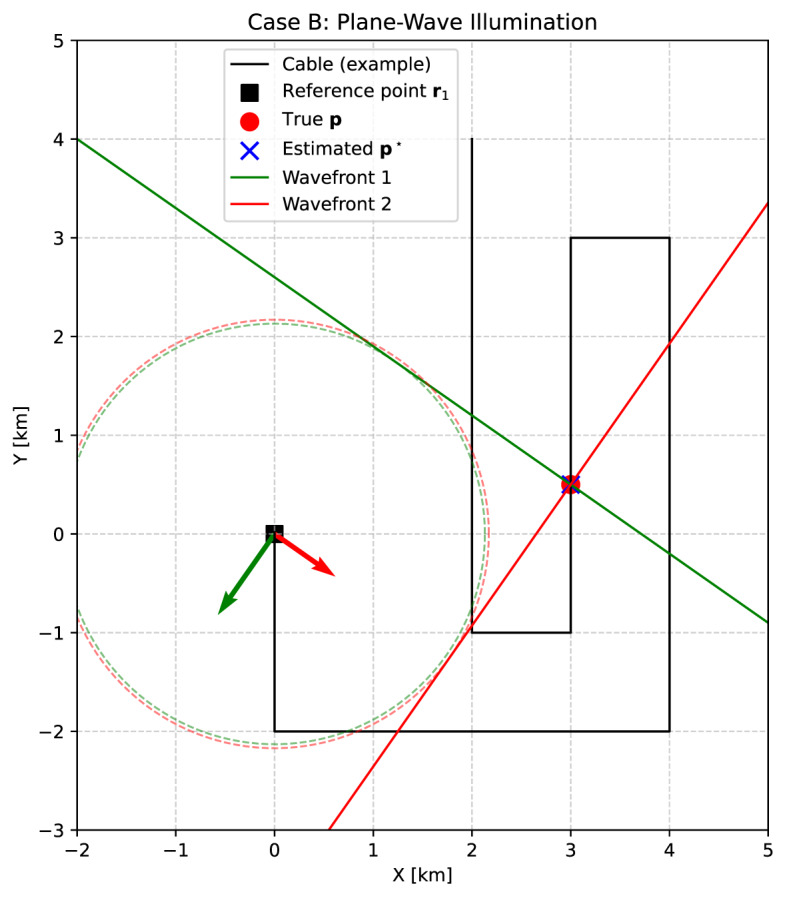
Localization of an unknown channel $\textbf{p}$ from plane-wave arrivals (Case B). The location of one reference point $\textbf{r}_1$ is known, and two plane waves with back azimuths $35^\circ$ and $-55^\circ$ and apparent velocities of $v=3.0$ km/s and $v=4.0$ km/s are assumed. Solid lines denote the reconstructed wavefronts, while dashed circles indicate distance constraints derived from the differential arrival times. Green and red arrows at the reference point illustrate the slowness vectors of the two wavefronts. For each plane wave, the corresponding wavefront can be constructed as the tangent to the circle in the direction defined by the slowness vector direction. The intersection of the reconstructed wavefronts, constrained by the arrival-time differences, defines the channel position; in practice, inconsistencies in the measurements lead to a misfit, and the estimated position is obtained by a least-squares solution.

This approach localizes an unknown channel $\textbf{p} = (x, y)$ along the fiber-optic cable using plane-wave arrivals. At least one reference point of known position, $\textbf{r}_1 = (x_1, y_1)$, is required. We consider two distinct plane waves, indexed by $m=1,2$, each characterized by a known and linearly independent slowness vector $\textbf{s}_m = (s_{xm}, s_{ym})$, i.e., the vectors are not parallel and provide independent directional constraints (Fig. 2).

Let $t_{im}$ denote the arrival time of plane wave *m* at reference point $\textbf{r}_i$, and $t_{pm}$ the corresponding arrival time at the unknown channel position $\textbf{p}$. The goal is to determine $\textbf{p}$ from the measured differential arrival times between the reference and the unknown locations.

If the slowness vectors are not known a priori, they can be estimated from arrival times at additional known points ($\textbf{r}_2, \textbf{r}_3$, etc.). Differences in arrival times at three or more non-collinear reference points allow for the reconstruction of the slowness vector of each plane wave, which can then be used in the localization procedure.

For a plane wave with a slowness vector $\textbf{s}_m$, the arrival time at $\textbf{r}_i$ is

6 $$\begin{aligned} t_{im} = \textbf{s}_m \cdot \textbf{r}_i + t_{0m}, \end{aligned}$$

where $t_{0m}$ is an unknown reference time. Subtracting the equations for $\textbf{r}_1$ and $\textbf{p}$ eliminates $t_{0m}$:

7 $$\begin{aligned} t_{pm} - t_{1m} = \textbf{s}_m \cdot (\textbf{p} - \textbf{r}_1). \end{aligned}$$

This yields a linear system for $\textbf{p} = (x,y)$:

8 $$\begin{aligned} \begin{pmatrix} s_{x1} & s_{y1} \\ s_{x2} & s_{y2} \end{pmatrix} \begin{pmatrix} x \\ y \end{pmatrix} = \begin{pmatrix} (t_{p1} - t_{11}) + \textbf{s}_1 \cdot \textbf{r}_1 \\ (t_{p2} - t_{12}) + \textbf{s}_2 \cdot \textbf{r}_1 \end{pmatrix}. \end{aligned}$$

Because $\textbf{s}_1$ and $\textbf{s}_2$ are linearly independent, the coefficient matrix is invertible, yielding a unique solution for (*x*, *y*). If more than two plane waves with independent slowness vectors are available, the system becomes overdetermined and can be solved in the least-squares sense, thereby improving robustness against arrival-time uncertainties.

#### Extension to unknown velocities and additional references

In practice, the apparent velocities of the plane waves may not be known with sufficient accuracy. In this case, a second reference point $\textbf{r}_2$ can be included. The arrival-time differences then provide constraints for the unknown location $\textbf{p}$ and for the unknown apparant velocity $v_m$ of each wavefront.

First, we decompose the slowness vector as

9 $$\begin{aligned} \textbf{s}_m = \frac{1}{v_m}\,\hat{\textbf{u}}_m, \end{aligned}$$

where $\hat{\textbf{u}}_m$ denotes the known propagation direction.

We then define the differential arrival times

$$\Delta t_{jim} = t_{jm} - t_{im},$$

such that the forward relation becomes

$$\Delta t_{jim} = \frac{1}{v_m}\,(\textbf{p}_j - \textbf{r}_i)\cdot \hat{\textbf{u}}_m.$$

Similar to case A before, a joint inversion for positions and apparent velocities can then be formulated by alternating between position and slowness updates. For fixed velocities, the channel positions are updated independently:

10 $$\begin{aligned} \textbf{p}_j^{(k+1)} = \arg \min _{\textbf{p}_j} \sum _{m,i} \left[ \Delta t_{jim} - s_m^{(k)} \, \big ( (\textbf{p}_j - \textbf{r}_i)\!\cdot \hat{\textbf{u}}_m \big ) \right] ^2, \end{aligned}$$

where k refers to the current iteration step. For fixed positions, each wavefront velocity is updated in closed form from all channels and references combined:

11 $$\begin{aligned} s_m^{(k+1)} = \frac{\displaystyle \sum _{j,i} \big [ (\textbf{p}_j^{(k+1)} - \textbf{r}_i)\!\cdot \hat{\textbf{u}}_m \big ]\, \Delta t_{jim}}{\displaystyle \sum _{j,i} \big [ (\textbf{p}_j^{(k+1)} - \textbf{r}_i)\!\cdot \hat{\textbf{u}}_m\big ]^2}, \qquad v_m^{(k+1)} = \frac{1}{s_m^{(k+1)}}. \end{aligned}$$

Equation (10) is applied independently to all channels for the current velocities, while Eq. (11) computes one global apparent velocity per plane wave by minimizing the misfit across all channels and references simultaneously.

Although this closed-form alternating update is efficient (and used in the numerical examples below), both position and velocity parameters can also be estimated together in a joint least-squares inversion. The solution can potentially be improved by inclusion of more than two plane waves or more than two reference points, if available.

Both approaches, Case A and Case B, provide independent means of localizing fiber-optic cable channels using DAS recordings. The following section presents synthetic results that illustrate the performance and limitations of the two methods.

## Results

The performance of the two localization approaches was assessed using synthetic tests. A bent cable geometry was defined as a 22 km long polyline consisting of several straight segments as shown in Fig. 1. Up to three reference points were assumed to be known along the cable at (0, 0), $(4000,-1000)$, and (2000, 1500). All other channels were placed at 200 m intervals (100 m in the heterogeneous case) along the cable and treated as unknowns.

### Case A: Ambient noise cross-correlation

Synthetic travel-time data were generated from the true cable geometry, assuming a propagation velocity of $v_\text {true} = 2000$ m/s. Gaussian noise with a standard deviation of 20 ms was added to the travel times $\tau _{i}$ (cf. Eq. 1) to mimic arrival-time uncertainties typical of ambient-noise interferometry. Localization was performed with a Levenberg–Marquardt least-squares algorithm that iteratively minimizes the misfit between observed and predicted distances.

We first perform the inversions with a prescribed propagation velocity. Three cases were considered: the true velocity (idealized baseline), a value 10% lower, and a value 10% higher, to illustrate the effect of systematic velocity mis-specification. To assess uncertainty, each unknown channel was re-localized 50 times with independently perturbed travel times, yielding approximately five thousand tests per scenario (107 channels $\times$ 50 realizations each). Each inversion run uses a single noisy realization of the travel times for a given channel. The spread in the recovered positions across the Monte Carlo ensemble thus quantifies the robustness of the method under noisy but fixed-velocity conditions. The inversion is initialized from the centroid of the reference points to provide a consistent starting value for all realizations.

**Fig. 3:**
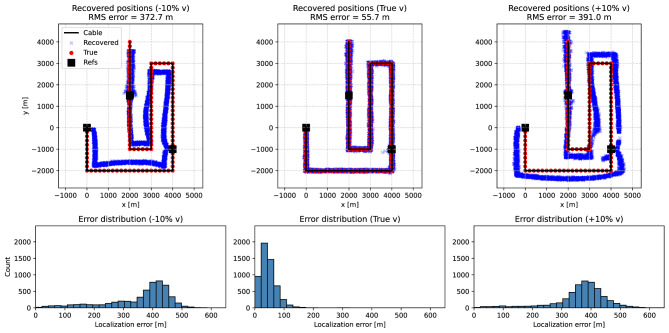
Synthetic Case A tests for three assumed propagation velocities: (left) 10% too low, (middle) true velocity, and (right) 10% too high. Top row: recovered channel positions (blue crosses), shown for 50 Monte Carlo realizations per channel, compared with the true geometry (red dots) and reference points (black squares). Bottom row: distributions of localization errors across all channels and all Monte Carlo realizations (50 noise perturbations per channel). Incorrect velocity assumptions introduce systematic biases in the recovered geometry, leading to increased localization errors. When the true velocity is used, the recovered positions closely match the true geometry, and the error distribution is significantly narrower.

Figure 3 summarizes the fixed-velocity tests. The top row shows the recovered channel positions compared to the true geometry (red dots) and reference points. The bottom row quantifies the corresponding localization errors across the Monte Carlo ensemble. A systematic bias is visible when the assumed velocity is incorrect. At the true velocity, the recovered positions scatter symmetrically around the correct locations, with uncertainties reflecting the added timing noise.

In a second test, the velocity was treated as an unknown common parameter (the global velocity), estimated jointly from all channels. The inversion was initialized from a value 10% higher than the true velocity, and refined iteratively using the alternating formulation described above: (1) channel positions are updated by local Levenberg–Marquardt steps, and (2) the global velocity is updated in closed form from all channel-reference pairs (as described above). In contrast to the fixed-velocity scenario, here only a single realization of noisy travel times was inverted. All channel positions were initialized from the centroid of the reference points, and the velocity update was applied with a relaxation factor to ensure stable convergence. For the geometry considered here, strongly offset starting positions can produce degenerate solutions, whereas the inclusion of (e.g.) a fourth well-placed reference point can restore accurate localization.

**Fig. 4:**
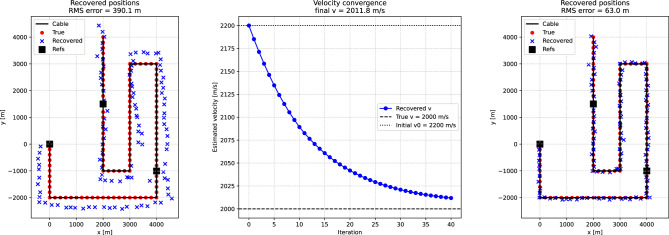
Global velocity inversion for Case A. Left: recovered channel positions using only the initial velocity guess ($v_0 = 1.1 \, v_\text {true}$). Middle: convergence of the velocity estimate (blue) toward the true value (dashed line), starting from the initial guess (dotted line). Right: recovered channel positions after joint inversion for geometry and velocity (blue crosses), compared with the true unknowns (red dots) and reference points (black squares) along the cable geometry (black line). Results are shown for a single noisy realization of the travel times, in contrast to the Monte Carlo ensemble used in the fixed-velocity tests (Fig. 3).

Figure 4 illustrates the performance of the global velocity inversion. The left panel shows the recovered channel positions when the initial velocity estimate ($v_0 = 1.1 \, v_\text {true}$) is used, leading to a significant misfit between true and recovered positions. The middle panel shows that the estimated velocity converges toward the true value within a few tens of iterations, despite the biased starting velocity. The right panel shows that the final recovered channel positions align closely with the true unknowns and the reference points, with no systematic offset along the cable geometry (black line). These results were obtained from a single noisy dataset, indicating that the algorithm can compensate for both travel-time noise and an initially biased velocity.

#### Ambient-noise based travel-time inversion in a heterogeneous velocity model

**Fig. 5:**
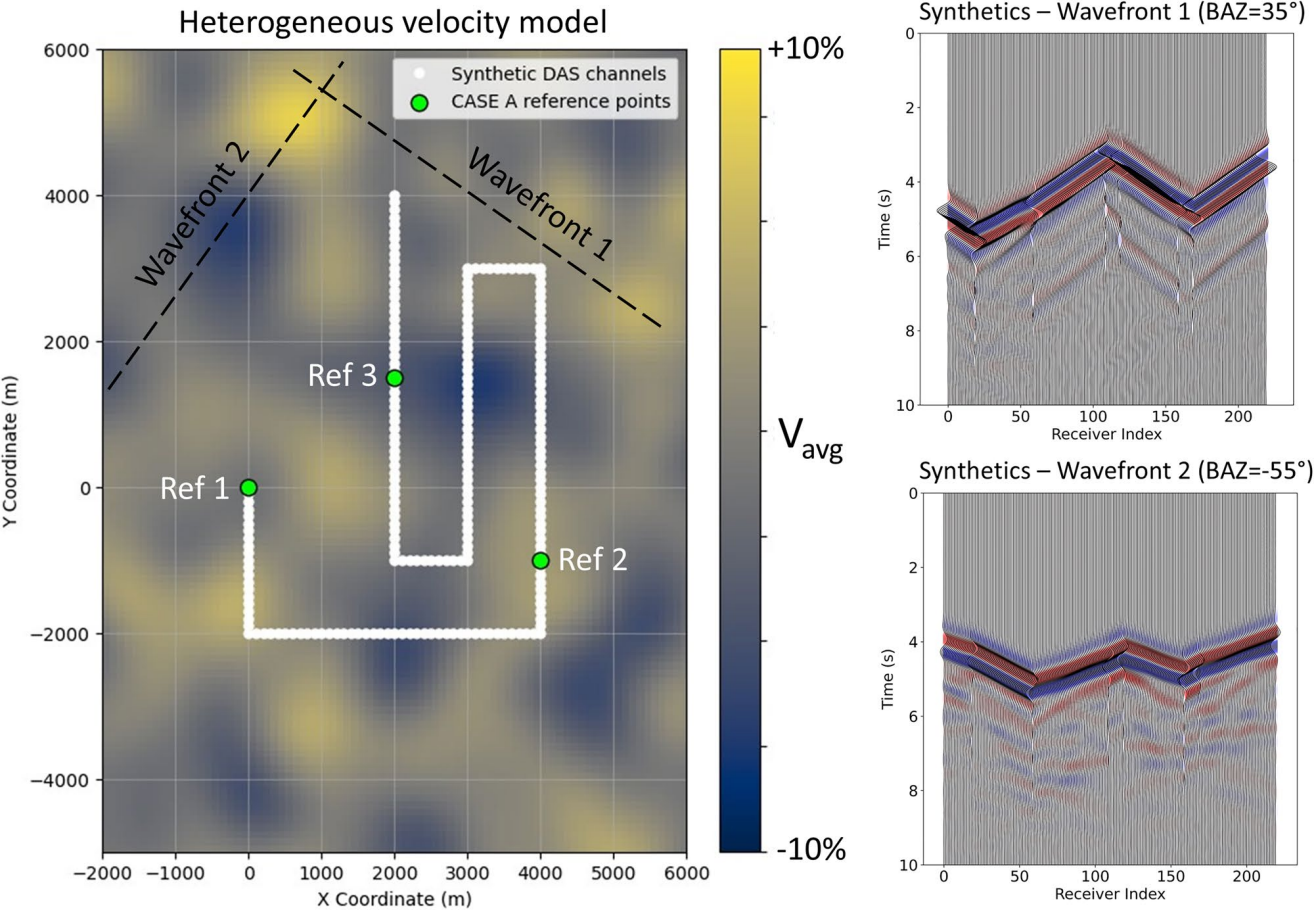
Left: Heterogeneous velocity model used for Case A and B simulations. The background color map shows $V_P$ with an average velocity of 2000 m/s and $\pm 10\%$ perturbations, corresponding to a dominant wavelength of about 1 km. The white polyline with dots at 100 m spacing represents the synthetic DAS cable geometry, with individual dots marking the channel positions. Green circles indicate the reference points (used in Case A) that serve as sources for the forward wavefield simulations. For Case B, simulations are performed using the same heterogeneous structure but with different propagation velocities (3000 m/s for $BAZ=35^{\circ }$ and 4000 m/s for $BAZ=-55^{\circ }$) to account for different apparant velocities for the plane wavefronts. Two reference channels at (0, 0) and $(4000,-1000)$ are used (see, e.g., Fig. 8). Right: Synthetic seismograms generated for two plane waves with backazimuths of $35^{\circ }$ and $-55^{\circ }$. Plane-wave propagation is simulated by distributing closely spaced sources along a line, producing a coherent wavefront traveling across the array. DAS directional sensitivity is accounted for by projecting particle motion onto the local fiber orientation. For the chosen geometry, incidence angles are close to $45^{\circ }$ along most of the cable, resulting in relatively uniform amplitudes and only minor directional effects.

To evaluate the performance of the localization and velocity inversion scheme under more realistic conditions, we generated synthetic seismograms representing Green’s functions in a two-dimensional heterogeneous velocity model (Fig. 5). The synthetic seismograms are computed using the spectral-element code Salvus^23^. The source is modeled as an isotropic point source with a Ricker wavelet of central frequency 1 Hz. The computational mesh uses four elements per minimum wavelength, ensuring adequate resolution of both the wavefield and the heterogeneous velocity structure. Absorbing boundary conditions are applied along the model boundaries to suppress artificial reflections. The model has an average velocity of 2000 m/s, superimposed with $\pm 10\%$ perturbations and a dominant wavelength of approximately 1 km. The reference positions along the cable were treated as sources, while the true channel positions served as receivers. For each source-receiver pair, synthetic wavefields were computed, and travel times (lag times $\tau$) were extracted by picking the first arrivals in the simulated seismograms. These delay times form the input for the inversion procedure.

The inversion (Case A) was initialized with a starting velocity that was 10% lower than the prescribed mean model velocity in order to test the ability of the algorithm to recover both the propagation velocity and the channel positions. All channel positions were initialized from the centroid of the reference points.

**Fig. 6:**
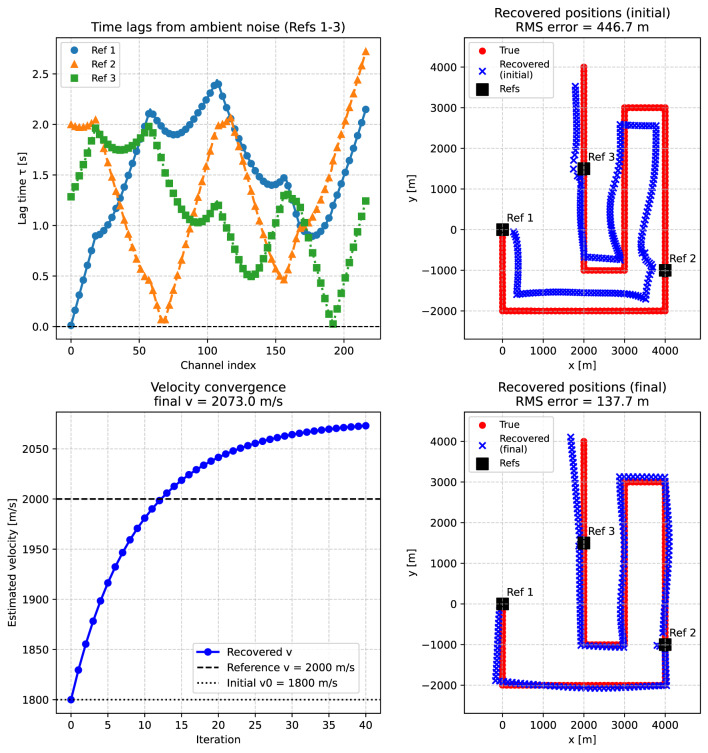
Inversion results for a heterogeneous medium using simulated ambient-noise recordings (extension of Case A). Top left: observed absolute lag times relative to the three reference channels. Top right: recovered channel positions obtained with the initial biased velocity estimate, leading to systematic deviations from the true geometry (red dots). Bottom left: convergence of the global velocity estimate (blue curve) toward an effective average velocity; the true average velocity of the heterogeneous model is indicated by the dashed black line. Bottom right: final recovered channel positions after joint inversion for geometry and propagation velocity (blue crosses), compared with the true unknowns (red dots) and reference points (black squares). Panel titles include the RMS localization error for the initial and final solutions.

The results are summarized in Fig. 6. The upper-left panel shows the observed lag times relative to the three reference channels, which exhibit coherent spatial trends along the cable and provide constraints for localization. The upper-right panel shows the channel positions recovered using only the initial velocity $v_0$, highlighting systematic offsets caused by the velocity mis-specification. The lower-left panel illustrates the convergence of the global velocity estimate: starting from the underestimated $v_0$, the recovered velocity (blue curve) stabilizes at a value slightly above the true model average (dashed black line). The lower-right panel presents the final recovered positions obtained after joint inversion for velocity and geometry, which align closely with the true receiver coordinates.

The inversion does not recover the true arithmetic average velocity of the heterogeneous model. It converges to an effective velocity that is biased toward a higher value. This overestimation arises because the inversion assumes a homogeneous medium, whereas the picked arrivals are controlled by the fastest propagation paths through the heterogeneous model. Therefore, the inversion velocity represents an apparent effective property of the wave propagation rather than the strict model average.

Quantitatively, the mean position error is significantly reduced compared to the initial solution, demonstrating that the joint inversion (partially) compensates for the biased starting velocity and for deviations introduced by the heterogeneous propagation model.

### Case B: Plane-wave illumination

In addition to the reference-based travel-time approach of Case A, we tested the localization of channels using synthetic plane-wave arrivals. In this scenario, starting with the homogeneous velocity case, only a single reference channel at (0, 0) is assumed to be known. Two plane waves with backazimuths of $35^{\circ }$ and $-55^{\circ }$ relative to north illuminate the array (see Fig. 2). The wavefronts were modeled with apparent velocities of 3000 m/s and 4000 m/s, which could correspond to P-wave phases that have turned within the crust before arriving at the array. For each plane wave, the arrival time at any channel is given by the projection of its coordinates onto the corresponding slowness vector. The differential travel times are then obtained as the arrival-time differences between the unknown channels and the reference. Gaussian noise with a standard deviation of 20 ms was added to the synthetic travel times to mimic uncertainty in the arrival-time picks. As timing errors are converted into spatial errors through the apparent velocity, the same 20 ms picking uncertainty results in larger localization scatter for faster plane waves.

We assume that the corresponding earthquake locations are known from localization with a conventional seismic network. In this case, the backazimuth to the unknown channels can be treated as well constrained, provided the sources are sufficiently distant such that the plane-wave approximation is valid. We therefore focus on the effect of velocity assumptions and also account for picking uncertainty. Localization was carried out by solving the linear system of equations that results from intersecting the wavefronts of the two plane waves. Three apparent-velocity scenarios were investigated: the correct values (3000 and 4000 m/s), a 10% underestimate for both wavefronts, and a 10% overestimate for both wavefronts. Synthetic data were always generated with the true 3000 and 4000 m/s apparent velocities, while the perturbed values were only used in the inversion. For each case, 50 Monte Carlo realizations were performed per channel, leading to ensembles of recovered positions and errors.

**Fig. 7:**
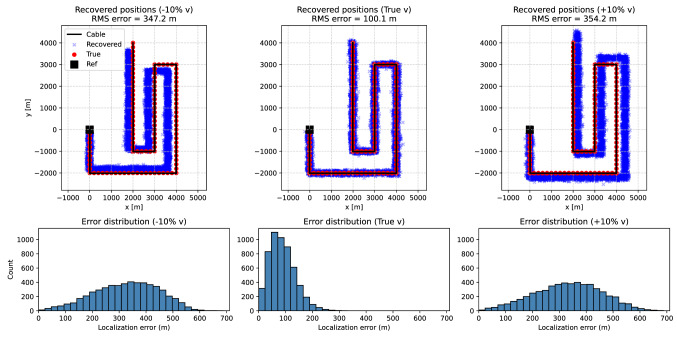
Synthetic Case B tests for two plane waves with backazimuths $35^{\circ }$ and $-55^{\circ }$ and apparent velocities of 3000 and 4000 m/s. Three inversion scenarios are considered: (left) both velocities 10% too low, (middle) correct apparent velocities, and (right) both velocities 10% too high. Top row: recovered channel positions (blue crosses), shown for 50 Monte Carlo realizations per channel, compared with the true geometry (red dots) and the known reference point (black square). Bottom row: distributions of localization errors across all channels and all Monte Carlo realizations (50 noise perturbations per channel). Incorrect velocity assumptions introduce systematic biases in the recovered geometry, leading to increased localization errors. When the correct apparent velocities are used, the recovered positions closely match the true geometry, and the error distribution is significantly narrower.

Figure 7 summarizes the results. The top row shows the recovered channel positions (blue crosses) compared to the true geometry (red dots) and the known reference point (black square). The bottom row shows the corresponding error distributions across all channels and Monte Carlo realizations. Similar to Case A, systematic biases arise when the assumed velocity deviates from the true value: underestimated velocities lead to recovered positions shifted toward the reference, while overestimated velocities push the solutions outward. When the correct velocity is assumed, the recovered positions scatter symmetrically around the true channel locations, with the spread reflecting the imposed timing noise. Overall, the test confirms that plane-wave illumination with two non-collinear azimuths is sufficient to reconstruct the cable geometry reliably, provided the apparent wavefront velocities are well constrained.

In a second experiment, we relaxed the assumption that the apparent velocities are known a priori. To solve the problem in this case, a second reference channel at $(4000,-1000)$ was introduced in addition to the one at (0, 0). This additional constraint makes it possible to estimate both the channel positions and the apparent velocities simultaneously. The inversion was formulated as an alternating least-squares problem, where channel positions are updated for given velocities, and velocities are then updated for the current positions (as described in the theory section). Starting from deliberately biased initial guesses ($v_1=2550$ m/s, $v_2=4600$ m/s), the algorithm converges within about 20 iterations to stable values close to the true inputs ($v_1=3000$ m/s, $v_2=4000$ m/s).

**Fig. 8:**
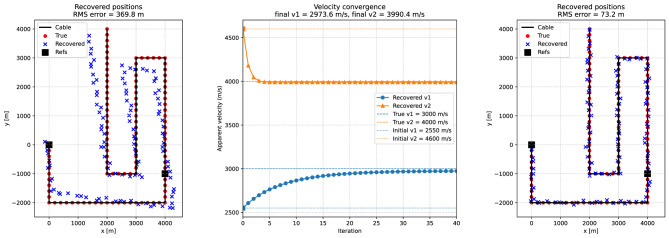
Global velocity inversion for Case B with two plane waves. Left: recovered channel positions using only the initial velocity estimates ($v_1=2550$ m/s, $v_2=4600$ m/s). Middle: convergence of the apparent velocity estimates (blue circles: wave 1; orange triangles: wave 2) toward the true values (dashed lines), starting from the initial estimates (dotted lines). Right: recovered channel positions after joint inversion of geometry and propagation velocities (blue crosses), compared with the true unknowns (red dots) and reference points (black squares) along the cable geometry (black line). Results are shown for a single noisy realization of the travel times, in contrast to the Monte Carlo ensemble used in the fixed-velocity tests (Fig. 7). Note that we use two reference points in the case of unknown apparant velocities.

Figure 8 illustrates the outcome. The left panel shows that, when the inversion is carried out with biased starting velocities, the recovered positions (blue crosses) deviate systematically from the true geometry (red dots). The middle panel demonstrates the velocity convergence: the apparent velocity for the first wavefront (blue circles) increases smoothly toward its true value, while it decreases for the second wavefront (orange triangles) from its initial overestimate, both approaching their target values within a few tens of iterations. The right panel shows the final geometry, where the recovered positions align closely with the true channel locations, and the systematic distortions have been removed. The residual scatter is consistent with the level of imposed timing noise (20 ms). This experiment demonstrates that Case B can be extended to situations where both geometry and apparent velocities are unknown.

#### Plane-wave-based travel-time inversion in a heterogeneous velocity model

To evaluate the performance of the plane-wave-based inversion scheme under realistic conditions, we repeated the Case B experiment in a heterogeneous velocity model analogous to that used in Case A (see Fig. 5). The two plane waves with backazimuths of $35^{\circ }$ and $-55^{\circ }$ were propagated through media whose local velocities vary by $\pm 10\%$ around nominal values of 3000 m/s and 4000 m/s, respectively. These variations mimic small-scale lateral heterogeneity and produce direction-dependent apparent velocities. The resulting delay times between the two reference channels at (0, 0) and $(4000,-1000)$ and all other fiber channels were extracted from synthetic wavefield simulations and served as input for the inversion.

As in the homogeneous case, both apparent velocities were treated as unknown parameters and estimated jointly with the channel coordinates. The inversion was initialized from deliberately biased starting values ($v_1=2500$ m/s, $v_2=4500$ m/s) and iterated using the alternating update scheme described above. In each iteration, channel positions were updated by least squares for the current velocity estimates, followed by closed-form least-squares updates of the apparent velocities.

**Fig. 9:**
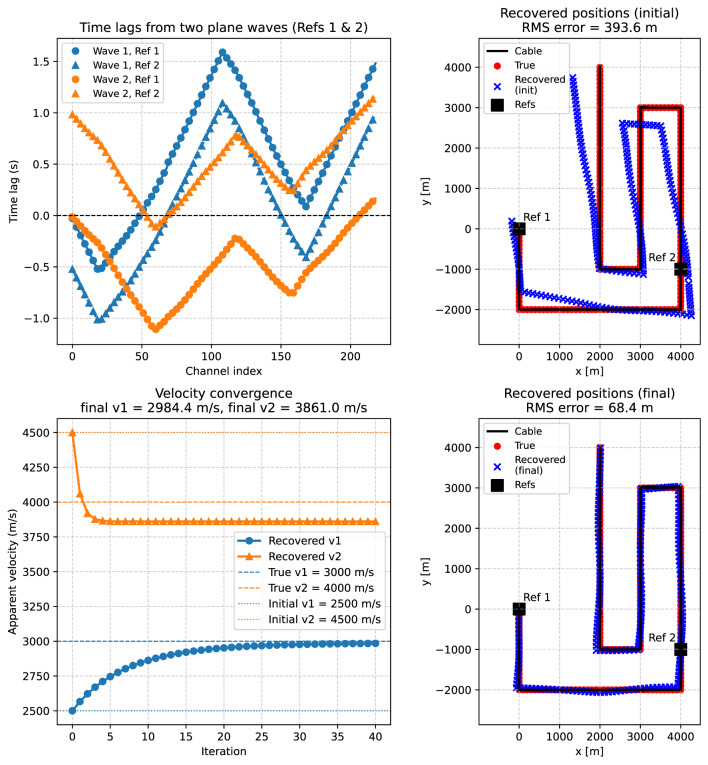
Inversion results for a heterogeneous medium using two plane waves with known backazimuths (extension of Case B). Top left: observed delay times relative to the two reference channels for both plane waves (wave 1 in blue, wave 2 in orange). Top right: recovered channel positions obtained with the initial biased apparent velocity estimates ($v_1=2500$ m/s, $v_2=4500$ m/s), leading to systematic deviations from the true geometry (red dots). Bottom left: convergence of the apparent velocity estimates for the two waves toward stable values; the true velocities are indicated by dashed lines. Bottom right: final recovered channel positions after joint inversion for geometry and propagation velocities (blue crosses), compared with the true unknowns (red dots) and reference points (black squares). Panel titles include the RMS localization error for the initial and final solutions.

Figure 9 summarizes the inversion results. When the inversion is initialized with biased apparent velocities of 2500 m/s and 4500 m/s, the recovered channel positions initially deviate systematically from the true cable geometry. The apparent velocities of both plane waves converge toward stable values within fewer than twenty iterations. After convergence, the recovered geometry reproduces the true channel locations with good accuracy (RMS error < 70 m), even in the presence of lateral velocity heterogeneity along the propagation paths.

Quantitatively, the joint inversion reduces the RMS position error from about 394 m to 68 m, demonstrating that the method effectively compensates for both the biased starting velocities and the influence of heterogeneous wave speeds. The remaining residual offsets indicate that the inversion assumes spatially constant velocities, whereas the synthetic data were generated in a spatially variable medium. Consequently, the estimated velocities represent effective apparent values along the dominant propagation paths rather than the true local wave speeds.

## Discussion

The results show that both approaches can be used to reconstruct fiber-optic cable geometry from DAS recordings using only a limited number of reference locations along the fiber. These findings support the potential of passive seismological methods for cable localization in settings where active surveys are impractical or not feasible.

However, the synthetic tests presented here rely on several simplifying assumptions. These include sufficiently reconstructed Green’s functions for travel-time estimates, the presence of coherent and approximately plane wavefronts, and relatively high signal-to-noise ratios to enable stable cross-correlation-based delay-time or lag estimation. These requirements further imply relatively smooth velocity variations and the absence of strong small-scale scattering. While these assumptions are necessary to investigate the performance of the proposed methods in the synthetic tests, they cannot fully account for the complexity of real DAS measurements. In particular, real-world wavefields may include reflected, refracted, and converted phases, as well as reverberations, which can interfere with lag estimation and, therefore, reduce localization accuracy.

Case A, based on ambient-noise interferometry (e.g., ^24,25^), requires at least three non-collinear reference channels to uniquely determine the channel positions. Synthetic tests indicate that the method remains robust for moderate levels of travel-time noise under the considered conditions. Biases caused by incorrect velocity assumptions can be significantly reduced when the propagation velocity is treated as an additional unknown. In heterogeneous media, the inversion converges toward an effective propagation velocity, which is sufficient to recover the cable geometry with an accuracy of $< 140$ m, although additional uncertainty is expected in more complex field settings.

Case B, in contrast, relies on plane-wave arrivals and requires only one reference channel, provided that two (or more) plane waves with independent azimuths are available^26^. This makes it applicable in cases where only limited reference information exists, such as offshore cable deployments^27,28^. Systematic errors arise when the apparent velocities are biased, but these can be reduced by introducing an additional reference channel and by joint inversion for positions and apparent velocities. The heterogeneous case further shows that both geometry and apparent velocities can be inferred simultaneously, yielding results with an accuracy of $< 70$ m. Real-world applications will likely lead to larger uncertainties.

Compared to fixed-velocity scenarios, joint inversion reduces biases induced by velocity mis-specification and yields consistent estimates of both geometry and propagation velocity. This highlights the importance of treating velocity as an unknown in realistic DAS localization problems, where the velocity structure is usually known only with limited accuracy.

### Practical considerations and limitations

While the synthetic tests provide insight into the performance of the methods, several factors relevant to real-world DAS data are not fully accounted for and are discussed in the following:

In both cases, the accuracy of the inferred geometry strongly depends on the reliability of the estimated time lags. These can be determined from the waveforms using semi-automatic approaches (e.g., cross-correlation between channels) or manual picking. Under low signal-to-noise ratio conditions, cross-correlation functions may exhibit multiple competing peaks, which can lead to cycle skipping and biased lag estimates. Similarly, for direct arrival picking, reduced amplitudes or interfering phases may increase picking uncertainty. These effects are not explicitly represented in the idealized synthetic tests and are expected to increase localization uncertainty in real-world applications.

DAS measurements are inherently directional, with recorded amplitudes depending on the angle between the incident wavefield and the local fiber orientation. For Case B, directional sensitivity can lead to spatial variations in signal amplitude, which in turn affect picking uncertainty. Segments of the cable that are less favorably illuminated (e.g., when the P-wavefield is oriented nearly perpendicular to the local fiber direction) will be affected by larger localization errors. In the geometry considered in this study, however, directional effects remain moderate due to favorable incidence angles (approximately $45^\circ$ relative to the orientation of the cable segments). This should be taken into account when selecting wave arrivals from different events.

For ambient-noise interferometry (Case A), directional sensitivity may be expected to have a smaller effect, as the reconstructed wavefield results from the superposition of scattered waves arriving from multiple directions. However, amplitude variations due to different fiber orientations at reference and unknown positions may still influence the signal-to-noise ratio of the correlations and thus the robustness of the lag estimates. These effects are not explicitly modeled here but should be considered when applying the method to field data.

The localization errors reported in this study should be interpreted independently of the channel spacing used in the synthetic examples. The relatively coarse spacing (100–200 m) was chosen for visualization purposes, whereas DAS measurements are typically available at much smaller spatial sampling intervals. Increasing channel density in the examples would not significantly change the absolute localization error but would provide a more detailed representation uncertainty field. However, the neighboring channels are expected to exhibit strongly correlated travel-time observations and therefore similar localization uncertainties. Interestingly, channel localization can potentially be improved further by incorporating the constraint that neighboring channels along the fiber are spatially close to one another.

In practical deployments, fiber-optic cables likely exhibit slack loops or strongly curved segments, which leads to discrepancies between along-fiber distance and true geographic position. In such cases, channels that are widely separated along the cable may be located in close spatial proximity. Methods that rely solely on along-fiber distance can therefore lead to misleading interpretations of cable geometry. The approaches presented here explicitly invert geographic positions and allow to resolve such effects. However, strongly curved geometries can present additional challenges, for example, due to the directionality of the recordings, as discussed above.

### Localization accuracy and possible improvements

The achieved localization accuracies of approximately $<140$ m (Case A) and $<70$ m (Case B) can be considered acceptable for many seismological analyses and monitoring applications, although specific requirements may vary^7,29^. While this accuracy is significantly lower than that of active calibration methods such as vehicle-tapping or hammer-impact surveys, which typically achieve meter- to decameter-scale precision but require on-site effort^4,13,18^, the passive approaches provide meaningful geometric constraints without external instrumentation, potentially over several tens to hundreds of kilometers.

We find that inversion results can also be sensitive to the choice of the initial starting point for the channel positions. In particular, unfavorable initial guesses may lead to convergence toward local minima, especially in cases with limited geometric constraints. This sensitivity can be reduced, for example, by choosing starting points near the centroid of the reference locations or by incorporating additional reference channels. Increasing the number of reference points strengthens the geometric constraints of the inversion and generally improves stability and robustness. The synthetic examples presented here deliberately use only the minimum number of reference points required for a unique solution, such that the reported performance represents a conservative estimate of the achievable accuracy.

In practice, the two methods can also be used in combination, for example by first using ambient noise to derive the geometry and by applying the plane-wave approach in a second step for refinement. A joint inversion using both methods represents another possibility. Both approaches benefit from additional constraints, such as additional reference points or wavefronts, which lead to overdetermined least-squares formulations and can enhance robustness against noise and modeling errors (e.g., ^30^). Further improvements may be achieved by integrating complementary information from OTDR, GPS-tracked sources, surface calibration measurements, or traffic noise.

## Conclusions

We presented two passive, seismology-based approaches for localizing fiber-optic cables with DAS: ambient-noise cross-correlation (Case A) and plane-wave illumination (Case B). Synthetic tests show that both methods are capable of recovering channel positions under controlled conditions and in the presence of realistic noise.

Both approaches benefit from the joint inversion for geometry and wave propagation properties. In Case A, the joint inversion of propagation velocity and geometry enables stable position estimates even in heterogeneous media, where the inferred velocity represents an effective property. In Case B, biases associated with uncertain apparent velocities can be overcome by including them as additional unknowns, leading to improved and more consistent localization results.

Overall, the results show the potential of passive DAS recordings to constrain cable geometry without requiring external surveys. While the achieved accuracy is lower than that of active calibration methods, it is sufficient for many seismological and monitoring applications. Future work should focus on validation using field DAS datasets and on extending the methodology to account for more complex wavefields and realistic measurement conditions.

## Data Availability

The software and numerical results generated during the current study are available from the corresponding author upon request.
